# Purification, Biochemical Characterization, and Bioactive Properties of a Lectin Purified from the Seeds of White Tepary Bean (*Phaseolus Acutifolius* Variety Latifolius)

**DOI:** 10.3390/molecules16032561

**Published:** 2011-03-21

**Authors:** Carmen Valadez-Vega, Ana María Guzmán-Partida, Francisco Javier Soto-Cordova, Gerardo Álvarez-Manilla, José A. Morales-González, Eduardo Madrigal-Santillán, José Roberto Villagómez-Ibarra, Clara Zúñiga-Pérez, José Gutiérrez-Salinas, Marco A. Becerril-Flores

**Affiliations:** 1 Institute of Health Sciences, Universidad Autónoma del Estado de Hidalgo, Ex-Hacienda de la Concepción, Tilcuautla, CP 42080 Pachuca de Soto, Hgo, Mexico; E-Mails: jmorales101@yahoo.com.mx (J.A.M.-G.); eomsmx@yahoo.com.mx (E.M.-S.); zupecl@yahoo.com.mx (C.Z.-P.); mbecerril_65@yahoo.com (M.A.B.-F.); 2 Center for Food Research and Development, A. C. Carretera a la Victoria Km 0.6 C.P. 83304. Hermosillo, Sonora, Mexico; E-Mails: gupa@ciad.mx (A.M.G.-P.); fsoto@ciad.mx(F.J.S.-C.); 3 Ezose Sciences Inc, Pine Brook, NJ 07058, USA; E-Mail: gmanilla@ezose.com (G.A.-M.); 4 Basic Science and Engineering Institute, Universidad Autónoma del Estado de Hidalgo, Carr. A-Pachuca-Tulancingo Km 4.5 Cd Universitaria, CP 42184, Mineral de la Reforma, Hgo, Mexico; E-Mail: roberto_ibarrav@hotmail.com (J.R.V.-I.); 5 Laboratory of Biochemistry and Experimental Medicine, Division of Biomedical Research, National Medical Center “20 de Noviembre”, ISSSTE, México D.F., Mexico; E-Mail: quauhtlicutli@yahoo.com (J.G.-S.)

**Keywords:** tepary bean, *Phaseolus acutifolius*, hemagglutinins, oligosaccharide specificity

## Abstract

The present work shows the characterization of *Phaseolus acutifolius* variety latifolius, on which little research has been published, and provides detailed information on the corresponding lectin. This protein was purified from a semi-domesticated line of white tepary beans from Sonora, Mexico, by precipitation of the aqueous extract with ammonium sulfate, followed by affinity chromatography on an immobilized fetuin matrix. MALDI TOF analysis of *Phaseolus acutifolius* agglutinin (PAA) showed that this lectin is composed of monomers with molecular weights ranging between 28 and 31 kDa. At high salt concentrations, PAA forms a dimer of 63 kDa, but at low salt concentrations, the subunits form a tetramer. Analysis of PAA on 2D-PAGE showed that there are mainly three types of subunits with isoelectric points of 4.2, 4.4, and 4.5. The partial sequence obtained by LC/MS/MS of tryptic fragments from the PAA subunits showed 90–100% identity with subunits from genus *Phaseolus* lectins in previous reports. The tepary bean lectin showed lower hemagglutination activity than *Phaseolus vulgaris* hemagglutinin (PHA-E) toward trypsinized human A and O type erythrocytes. The hemagglutination activity was inhibited by *N*-glycans from glycoproteins. Affinity chromatography with the immobilized PAA showed a high affinity to glycopeptides from thyroglobulin, which also has *N*-glycans with a high content of *N*-acetylglucosamine. PAA showed less mitogenic activity toward human lymphocytes than PHA-L and Con A. The cytotoxicity of PAA was determined by employing three clones of the 3T3 cell line, demonstrating variability among the clones as follows: T4 (DI_50_ 51.5 µg/mL); J20 (DI_50_ 275 µg/mL), and N5 (DI_50_ 72.5 µg/mL).

## 1. Introduction

Some legumes are a particularly rich sources of lectins; the bulk of these molecules are located in protein bodies inside the cotyledon of the seeds, although they have also been found in leaves, stems, and roots. The lectin concentration can be up to 10% of the total nitrogen content of the mature seed [1,2,3]. Lectins from different species of legumes have similar molecular properties [4]. For example, all of them are oligomeric glycoproteins composed of two to four subunits, each with one carbohydrate binding site. Many legume lectins are metalloproteins, which require divalent cations (Ca^++^, Mg^++^, or Mn^++^) for their biological activity. The amino acid sequences of the lectins from different legume species exhibit homologies ranging from 30 to 90% [5]. Additionally, legume lectins share common structural features, such a characteristic jelly-roll motif in their tertiary structure and similar oligomerization patterns [5,6]. Despite these similarities, lectins differ markedly in their carbohydrate binding specificity [3,7,8]. The majority of the identified legume lectins can agglutinate a variety of plant and animal cells, such as erythrocytes, lymphocytes, and malignant cells [3,4,9]. Lectins can be used to identify and purify a wide variety of polysaccharides, glycoproteins, and glycolipids [10,11].

The lectins from the common bean *Phaseolus vulgaris* have been identified and characterized in cultivars from all over the world [12]. The most extensively characterized lectins from this species have been purified from the “red kidney” variety. The lectin fraction from this bean is composed of five kinds of isolectins, each consisting of non-covalently bound tetramers made up of different combinations of subunits, which are known as E (erythroagglutinating) and L (leukoagglutinating). Each of these subunits differs from the other slightly in their amino acid sequences and possesses differential affinities for erythrocytes and lymphocytes [13,14,15,16,17,18].

The tepary bean *Phaseolus acutifolius* A. Gray is an annual legume adapted to arid and semi-arid regions extending from North America to Costa Rica, including Puerto Rico and Mexico. Tepary beans thrive under adverse agronomic conditions such as high salt concentrations and low water levels. Additionally, this species possesses high resistance to microbial pathogens and other predators [19,20,21]. Like other legume beans of the genus *Phaseolus*, tepary beans produce lectins and other anti-nutritional factors [22,23,24]. The tepary bean is quite toxic to man and animals in its raw form due to the presence of lectins in its seeds. There are some reports concerning lectins purified from tepary beans cultivated in different places. Some of these works mention the high quantities of amino acids, such as aspartic acid, serine, threonine, leucine, phenylalanine, asparagine, glutamic acid, and glycine [25,26]. The molecular weight of purified lectins from tepary bean from Puerto Rico was reported to be 83 kDa, while for lectins from a Chiapas (Mexico) variety it was 117 kDa, and the isoelectric points were 4.5 and 5.5, respectively. Another study reported a molecular weight in the range of 115–120 kDa for the tetrameric lectins purified from tepary beans from Queretaro, Mexico [27]. In this paper, we report on the purification, biochemical characteristics, and bioactive properties of white tepary bean lectins.

## 2. Results and Discussion

### 2.1. Isolation of Lectins

**Figure 1:**
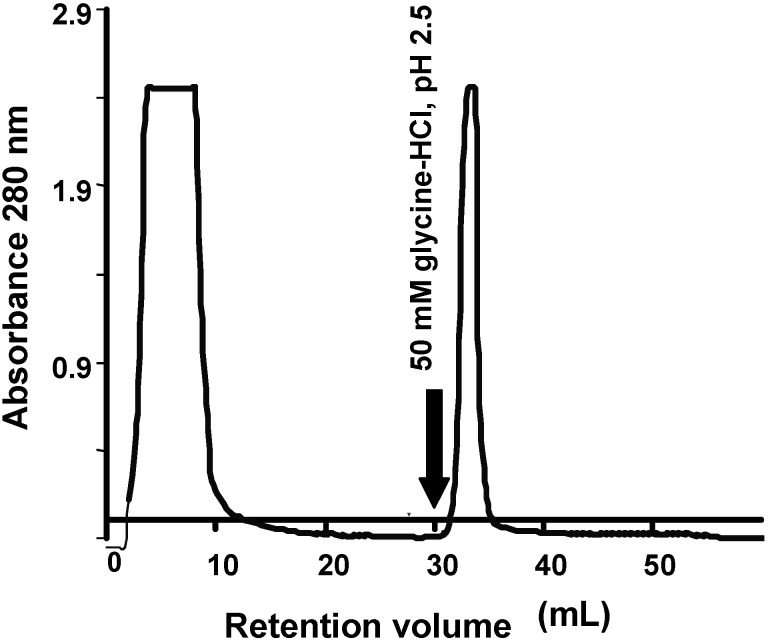
Affinity chromatography in immobilized fetuin of the aqueous extract from tepary beans after precipitation with ammonium sulfate. The lectin fraction was eluted with an acid solution (50 mM glycine-HCl, pH 2.5). The purification yield is shown in Table 1.

The tepary bean lectin was purified by affinity chromatography after ammonium sulfate precipitation of the aqueous extract from tepary beans (Figure 1). Tepary bean crude extract had a protein concentration of 30.418 mg/mL, with a titer of 262,144 units and hemagglutination activity of 8,618.05 units/mg per mL protein.

The lectin fraction was recovered in one peak when the affinity column was eluted with glycine 50 mM, pH 2.5; this fraction showed a titer of 26,214.4 units, with hemagglutination activity of 226,376.51 units/mg per mL protein, with a 26-fold purification achieved at this point (Table 1).

**Table 1:** Purification table of the lectin from tepary beans (*Phaseolus acutifolius*).

Fraction	Protein Concentration (mg/mL)	Hemagglutination Titer *	Specific Activity	Purification factor	Lectin (%)
Crude Extract	30.41	262144	8618.05	1	
Protein bound to fetuin	1.16	262144	226376.51	26	0.586

* Human A Erythrocytes trypsinized.

Differences in hemagglutination activity was observed with human erythrocytes from blood type A, B, and O (data not shown), indicating that PAA presents blood group specificity, contrary to what was observed for tepary bean variety escumite, in which there was no blood group specificity [28].

The unbound fraction was applied to the fetuin affinity column until no binding was observed. Analysis of this unbound fraction showed that it retained high hemagglutination titers (data not shown). This result suggests that there are other fractions with hemagglutination activity that do not demonstrate affinity for fetuin. Therefore, this study was focused on the description of the fraction that showed affinity for fetuin.

**Figure 2:**
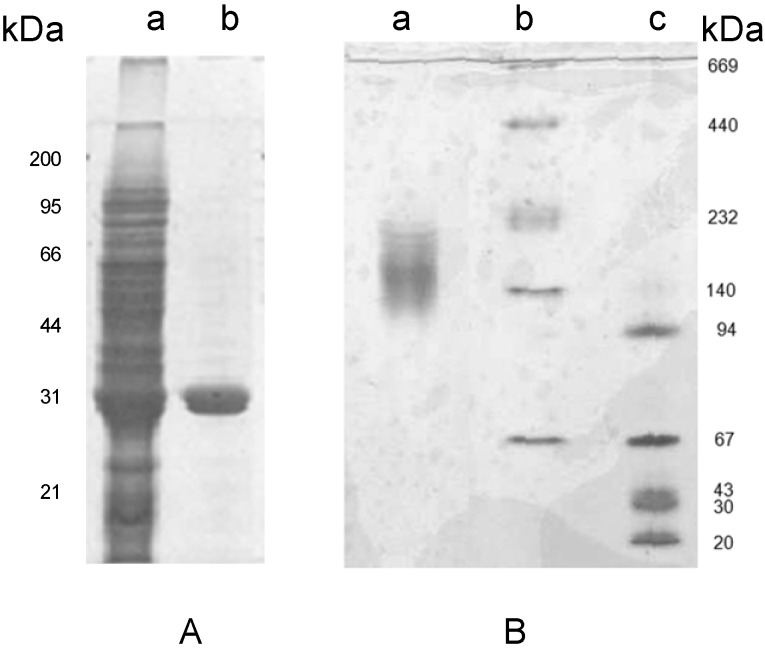
Electrophoretic analysis of the crude extract and pure lectin fractions from tepary beans on SDS-PAGE (Panel A) and Native PAGE (Panel B). Lane a in Panel A corresponds to the crude extract, lanes b and c corresponds to the pure lectin subunits with a molecular mass of 31 kDa. Panel B, line a shows that the lectin forms oligomers between of 132 and 153 kDa; lines b and c show the native Mw standards.

Figure 2A depicts the electrophoretic pattern of the fetuin-bound fraction on SDS-PAGE under reducing conditions. This pattern showed a single band indicating that the hemagglutinating protein was purified to homogeneity.

### 2.2. Chemical Characterization of the Lectin

Table 2 shows the amino acid composition results, which were determined by acid hydrolysis of the protein and subsequent *O*-phtalaldehyde derivatization. The amino acid content results showed differences with those reported by other authors for other varieties of tepary beans, because they found higher concentrations of Asx, Ser, and Leu [26,28,29], whereas in PAA the amino acids Gly, Leu, Met, and Ile, were found at higher concentration, and Lys, Glx, and Ala at a low concentration.

**Table 2:** Amino acid composition of the tepary bean lectin.

Amino Acid	ng/mg	%
Lys	10.91	0.24
Glx	21.25	0.47
Ala	34.53	0.77
Val	52.22	1.17
His	109.50	2.45
Tyr	190.64	4.26
Arg	190.92	4.27
Phe	257.31	5.75
Asx	269.82	6.03
Ser	347.55	7.77
Thr	396.12	8.85
Ile	401.79	8.89
Met	460.67	10.29
Leu	472.22	10.55
Gly	1259.25	28.14
Cys	n.d.^a^	n.d.

^a^ n.d. not detected.

Analysis of the pure lectin by the phenol sulfuric assay showed that lectin from tepary beans contains 6.5% of carbohydrates. These sugars are most likely part of *N*-linked oligosaccharides, as is the case of most legume lectins [30,31]. The studies indicated that PAA is a glycoprotein that also contains metal ions in its structure, and various studies have shown that these structural components are of major importance for the biological activity of lectins [29,32,33,34].

Table 3 shows the concentration on metals, in parts per million (ppm), where it was observed that calcium was found at a higher concentrations than the other metals, while chromium was the one with the lowest concentration, and cadmium was not found in the lectin. Despite the presence of considerable amounts of calcium, no inhibition of hemagglutination activity was observed when EDTA was added to the hemagglutination reaction at concentrations of 100 mM (data not shown). 

**Table 3:** Metal content of PAA determined by plasma spectrometry.

Metal	Concentration (ppm)
Ca	10739.9
Cu	2528.8
Zn	847.3
Mg	453.8
Fe	324.6
Mn	240.5
Cr	19.8

### 2.3. Oligomerization of the Lectin

**Figure 3:**
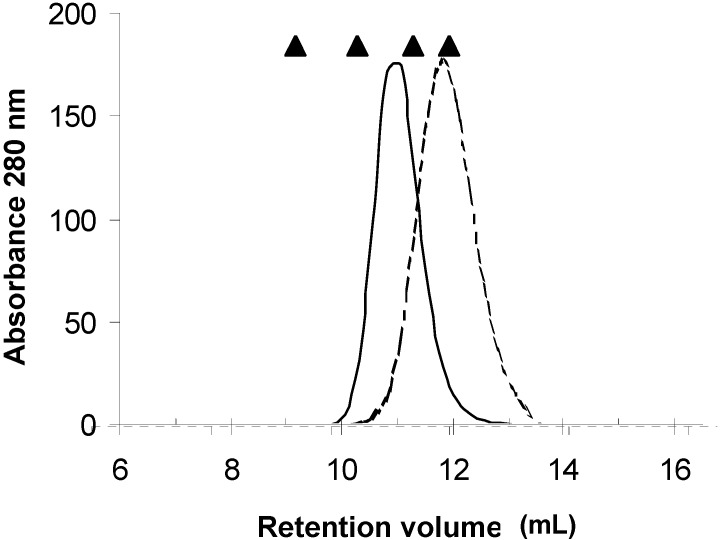
Analysis of oligomerization of the subunits of PAA by size exclusion chromatography on a superose 12 column (1.0 × 30 cm). Pure lectin was injected into the column and eluted at low ionic strength (—) and 1 M NaCl (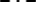). Elution positions of the molecular weight standards (Thyroglobulin 660 kDa, aldolase 150 kDa, bovine serum albumin 67 kDa, and ovoalbumin 44 kDa) are indicated (▲).

Separation of PAA by SDS-PAGE (Figure 2A) showed a single band with a molecular weight (Mw) of 31 kDa, whereas analysis by native gel electrophoresis showed a broad band in the range between 132 to 153 kDa (Figure 2B). Analysis of this lectin by MALDI-TOF revealed the presence of two species with Mw of 28 and 31 kDa, suggesting that the matrix used with fetuin has affinity for at least two isolectins, and that it forms an oligomer of at least four subunits. To confirm the results on the oligomerization of the lectin, one aliquot of the purified protein was injected into a TSK 3000-SW size exclusion chromatography column using PBS as elution buffer (data not shown). This analysis showed that PAA has an Mw of 62 kDa for the oligomeric protein, which suggests, in contrast to the native PAGE data, that it behaves like a dimer. To resolve these contradictory results, the role of ionic strength in the oligomerization of the lectin was tested. For this purpose, a set of size exclusion chromatography runs was performed in the lectin fraction using a Superose 12 size exclusion chromatography column (Figure 3).

In the first run, protein was eluted with a buffer containing 25 mM MES pH 6.0. This run yielded an Mw estimation of 123 kDa, corroborating the tetrameric behavior of the oligomeric lectin. However in a second run, when 1 M NaCl was added to the MES buffer, the estimated Mw was 63 kDa (close to a dimeric behavior). These data demonstrated that the concentration of salt affected the oligomerization of the lectin. It is known that legume lectins can form oligomers consisting of two or four subunits. Although many of the structural features of the oligomerization of legume lectins have been elucidated [30], there is still little information on the details of the mechanism for the oligomerization process. There are few studies on the influence of the ion concentration in the oligomerization process of any lectin. Hatakeyama *et al*. and Kuwahara *et al*. [35,36] reported that high concentrations of NaCl (1-M) and high pH values (9–10) induce oligomerization of the lectin CEL-III from the sea cucumber *Echinaria cucumata* when small carbohydrate ligands are present in the lectin solution. In the present study, low concentrations of NaCl favor the formation of a tetramer, as opposed to the formation of a dimer at higher salt concentrations. The previously noted results allowed us to conclude that the lectin isoforms found in these beans are composed of four subunits, which agrees with reports for other lectins from other varieties of tepary beans [26,29,37].

### 2.4. Characterization of the Subunits of the Tepary Bean Lectin

**Figure 4:**
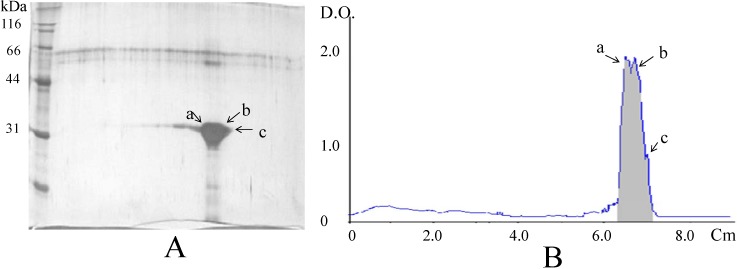
Analysis of the lectin from tepary beans by 2D-polyacrylamide gel electrophoresis. (**A**). In the gel shown, three distinct species (designated as a, b, and c) were distinguished; (**B**). Densitogram shows peaks corresponding to the three species, with calculated isoelectric points of 4.5 (a), 4.4 (b), and 4.2 (c).

Analysis of the lectin fraction by 2D-PAGE resulted in the separation of three protein species with the same molecular weight but different isoelectric points (4.2, 4.4, and 4.5) (Figure 4); each subunit is assumed to correspond to a different subunit. 

Further analysis of the amino acid sequences of six tryptic peptides from these subunits (Table 4) showed that all of them share between 90 and 93% of identity with erythroagglutinating phytohemagglutinin, leukoagglutinating phytohemagglutinin from *Phaseolus vulgaris* [38], and phytohemagglutinin from *Phaseolus coccineus* [39], and 100% identity with phytohemagglutinin from *Phaseolus acutifolius* [40]. This latter sequence was isolated when a c-DNA from tepary beans was screened with a probe derived from the sequence of the α-amylase inhibitor from *Phaseolus vulgaris*. Mirkov *et al*. [40] did not report on this sequence, nor did they purify a lectin fraction from tepary beans. Therefore, the present work confirms that the sequence reported in that study corresponds to an active lectin. Due to the identity found among the sequences of other lectins, as mentioned previously, and the subunits separated by 2D-PAGE for PAA, it is suggested that the differences in pI in the resolved species are due to post-translational modifications, such as glycosylation.

**Table 4:** Results of the sequence obtained by LC/MS/MS on tryptic peptides of proteins excised from 2D-PAGE separation of the tepary bean subunits. The calculated masses were obtained from a theoretical tryptic digest of the sequence of the lectin precursor from *Phaseolus acutifolius* reported in the NCBI protein sequence data base (Accession No gi|1086123).

Calculated Mass	Observed Mass	Start residue	End residue.	Sequence
1095.6156	1095.6118	161	170	HIGIDVNSIK
1317.7776	1317.7792	197	208	LLVASLVYPSQK
1324.6995	1324.7072	209	220	TSFIVSDTVDLK
1635.8125	1635.7968	140	153	AHTVAVEFDTLYNR
1800.9086	1800.8922	56	72	LTNLNDNGEPTLSSLGR
2219.2183	2219.2234	102	124	VPNNAGPADGLAFALVPVGSKPK

### 2.5. Hemagglutination Activity

Table 5 shows a comparison of specific activity on agglutination assays of the tepary bean hemagglutinin with commercial lectin from *Canavalia ensiformis* (concanavalin A). Our results showed that, in general, tepary bean hemagglutinin had higher activity than concanavalin A. When hemagglutination assays were carried out with non-trypsinized erythrocytes, it was observed that hemagglutination activity was lower than when erythrocytes were pretreated with trypsin s (data not shown).

**Table 5:** Hemagglutination activity of the purified lectin from *Phaseolus acutifolius* compared with that of the lectin from *Canavalia ensiformis* (Concanavalin A) on trypsinized human erythrocytes of blood types A and O.

Lectin	Lectin Concentration (mg/mL)	Trypsinized human erythrocytes(Hemaggluting titer)	
		Type A	Type O
Tepary bean	3.5	292.6	36.6
*Phaseolus vulgaris* (PHA-E)	3.5	20.34 × 10^7^	33.55 × 10^6^
Concanavalin A	3.5	1.1	0.57

The effect of various monosaccharides, oligosaccharides, and glycoconjugates on hemagglutination activity was tested in the present study. These assays were performed in 96-well microtiter plates in which the concentration of the potential inhibitor was varied and the lectin concentration was maintained constant.

The results obtained in these experiments (Table 6) showed that monosaccharide, oligosaccharides, and glycopeptides did not any inhibitory effects on the hemagglutination activity. However, on the other hand, intact glycoproteins showed an inhibitory effect in erythrocytes of both types A and O.

**Table 6:** Effect of glycans and glycoconjugates on the hemagglutination activity of the lectin from tepary beans.

	Inhibitory concentration ^a^ (mg/mL)	
**Glycoproteins**	Human “O” Erythrocytes	Human “A” Erythrocytes
GnT-V	1.7 × 10E-3	27.3 × 10E-3
Fetuin	78.1 × 10E-3	156.3 × 10E-3
Fibrinogen	9.8 × 10E-3	39.1 × 10E-3
Thyroglobulin	4.8 × 10E-3	4.8 × 10E-3
Ovoalbumin	1.3	1.3
**Glycopeptides**		
Tryptic glycopeptides from bovine fetuin	n.i.	n.i.
Tryptic glycopeptides from pocine Thyroglobulin	n.i.	n.i.
Tryptic glycopeptides from ovoalbumin	n.i.	n.i.
Thermolytic glycopeptides from Fibrinogen	n.i.	n.i.
**Oligosaccharides**		
Chitooligosaccharides	n.i.	n.i.
Human milk oligosaccharides mixture	n.i.	n.i.
Raffinose	n.i.	n.i.
Lactose	n.i.	n.i.
**Monosaccharides**		
Maltose	n.i.	n.i.
Glucose	n.i.	n.i.
Galactose	n.i.	n.i.
Mannose	n.i.	n.i.
Fucose	n.i.	n.i.
Metilglucopyranose	n.i.	n.i.
Glucosaminitol	n.i.	n.i.
Galactosaminitol	n.i.	n.i.

^a^ lowest concentration giving complete inhibition.

These results indicate that *N*-linked complexes (biantennary triantennary, or tetraantennary) are the best haptens for this lectin because these structures are those that present in the glycoprotein inhibitors that rendered the best inhibition. A summary of the structures that are present in the glycoproteins that were utilized in the hemagglutination inhibition assays is presented in Table 7. Bovine fetuin expresses mainly biantennary structures [40]. Triantennary oligosaccharides are the main components of bovine fetuin [41]. Porcine thyroglobulin possess a variety of biantennary and triantennary *N*-linked glycans [42], and the recombinant human β-1,6-*N*-acetylglucosaminyltransferase-V used in this study contains a variety of biantennary, triantennary, and tetraantennary glycans [43]. PAA, however, may have low affinity for high mannose and hybrid structures because these are the structures present in ovoalbumin [44] and because this glycoprotein caused moderate inhibition of the hemagglutinating reaction. The fact that glycopeptides that were prepared from the glycoproteins mentioned previously did not cause inhibition of the hemagglutinating reaction is an indication that cooperativity (clustering of multivalent ligands) is important in the binding of PAA to its ligands [45]. Therefore, these results suggest that PAA, like other lectins [28,46,47,48] also belong to the group termed “complex” with specificity toward *N*-glycans.

**Table 7:** Asparagine-linked oligosaccharide structures that are present in the glycoproteins and glycopeptides utilized in inhibition of the hemagglutination assays of PAA.

Structure	Name	Source(s)	References
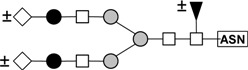	Biantennary complex	Bovine Fibrinogen	[36,40,49,50]
		Bovine fetuin	
		Porcine Thyroglobulin	
		GnT-V	
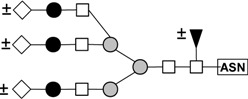	Triantennary complex	Bovine fetuin	[40,49,50]
		Porcine Thyroglobulin	
		GnT-V	
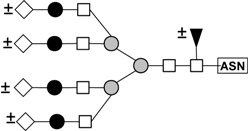	tetrantennary complex	GnT-V	[49]
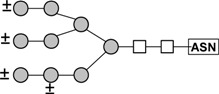	High mannose	Ovoalbumin	[40,41]
		Porcine Thyroglobulin	
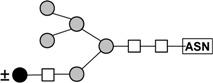	Hybrid	Ovoalbumin	[40,41]
		Porcine Thyroglobulin	

● Galactose; □ *N*-Acetylglucosamine; 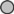 Mannose; ◊ *N*-Acetylneuraminic (sialic) acid; ▲ Fucose; **±** indicates that the monosaccharide residue may or not be present as part of the structure.

**Figure 5:**
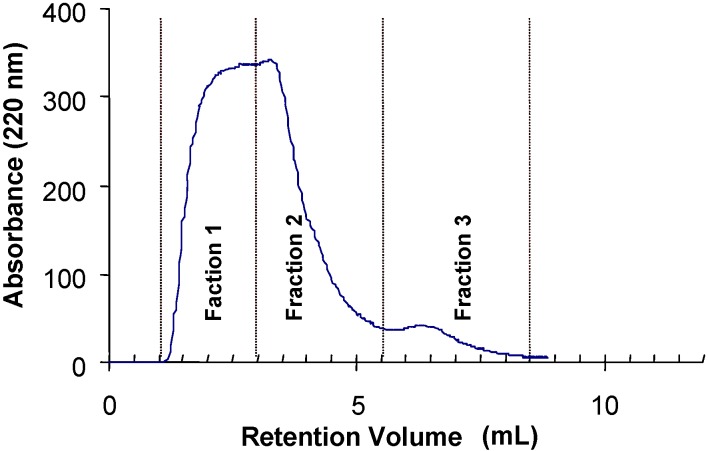
Analysis of the carbohydrate specificity of tepary bean hemagglutinin by lectin affinity chromatography. A glycopeptide mixture purified from thermolysin-treated thyroglobulin was applied to a column with a resin containing immobilized PAA. The glycopeptides were eluted in three fractions. Fraction 1 contains the unbound glycopeptides. Fractions 2 and 3 are the fractions that were retarded by the lectin column and that therefore have affinity for the lectin. Carbohydrate composition of these fractions is presented in Table 8. The proposed structure of the oligosaccharide with the highest affinity with the tepary bean lectin is presented in Figure 6.

The binding of glycopeptides from porcine thyroglobulin with a high variety of complex *N*-glycans showed that some of these glycopeptides were retarded by the immobilized tepary bean lectin. The chromatogram depicted in Figure 5 shows three distinct fractions: fraction 1 corresponds to the unbound (run-through) material, and fractions 2 and 3 are the glycopeptides that were retarded by the affinity column. The monosaccharide composition of the three fractions from lectin affinity chromatography presented in Table 8 shows that the retarded glycopeptides contained in fraction 3 have a larger proportion of *N*-acetylglucosamine in their structures.

**Table 8:** Carbohydrate compositions of the glycopeptide pools separated by affinity chromatography in an immobilized PAA column (Figure 6).

Carbohydrate Residue	Monosaccharide composition (%)		
	Fraction 1	Fraction 2	Fraction 3
Fucose	5.8	5.5	3.9
Mannose	49.1	43.8	26.0
Galactose	15.5	14.9	15.8
*N*-acetylglucosamine	22.6	29.6	40.5

**Figure 6:**
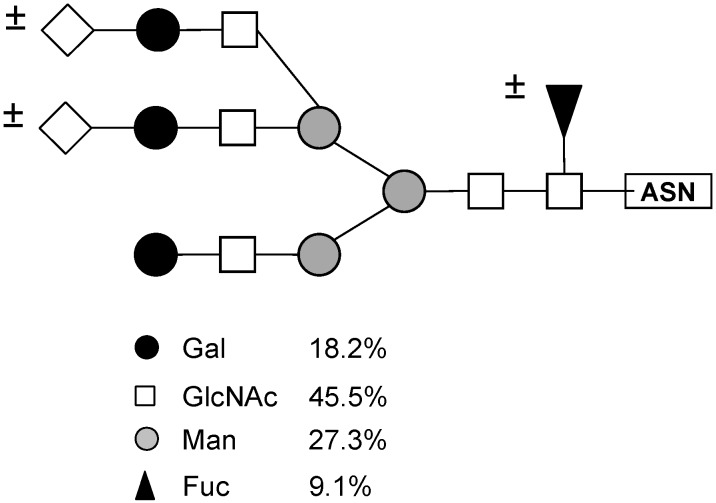
Structure of the oligosaccharide from porcine thyroglobulin (Figure 5, Fraction 3), which has the highest affinity to the lectin from tepary beans. This structure was proposed based on the monosaccharide composition of this fraction, shown in Table 8.

Based on this monosaccharide composition, the structure shown in Figure 6 (a desialylated triantennary structure lacking a galactose residue in one of its branches) is proposed as the best ligand for PAA in the glycopeptides mixture from thyroglobulin.

### 2.6. Mitogenic Activity

The mitogenic activity of PAA was compared with that of the leukoagglutinating lectin from the common bean lectin (PHA-L) and concanavalin (Con A). These assays were carried out by measuring the amount of [^3^H] thymidine that was incorporated into cultured human lymphocytes at various doses.

**Figure 7:**
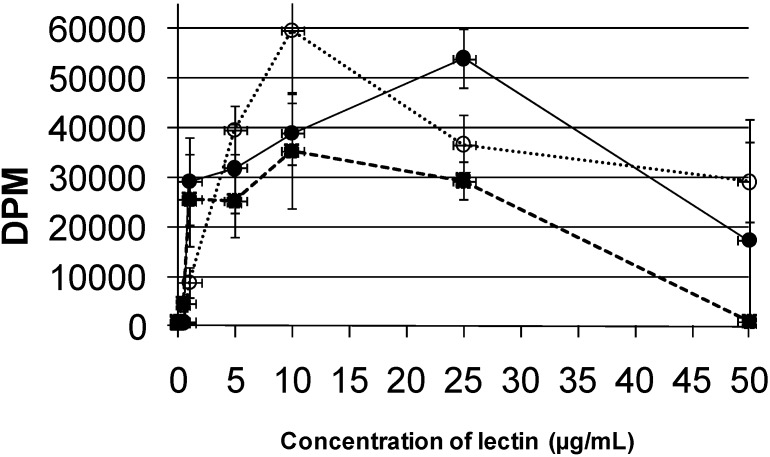
Comparison of the mitogenic effects of the lectin from tepary beans (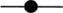), the leukoagglutinating lectin from *Phaseolus vulgaris* (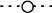), and Concanavalin A (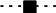). In these experiments, the incorporation of [^3^H]-thymidine was measured after incubation of cultured human lymphocytes with increasing amounts of each lectin.

Results on the mitogenicity of the assayed lectins (PAA, PHA-L, and Con A) are presented in Figure 7. These results indicate that the lectins from PHA-L and Con A possess higher potential for stimulation of cell division, showing their maximum mitogenic effect at concentrations of 10 μg/mL; on the other hand, PAA showed its maximum mitogenic effect at 25 μg/mL. Studies with *Phaseolus acutifolius* variety escumite lectin [28] showed the same mitogenic capacity as the PAA, while, on the other hand, Vargas-Albores *et al.* [26] reported that a lectin tepary had greater mitogenic activity than that of PAA.

### 2.7. Cytotoxicity Activity on Mouse 3T3 Fibroblast Cell Clones

**Figure 8:**
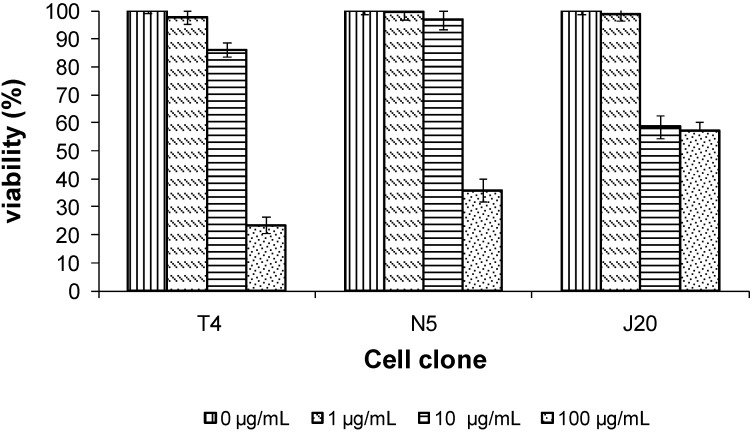
Effect of the lectin from tepary beans on mouse 3T3 fibroblast cell clones. Cell clones were exposed to the indicated concentration of tepary bean lectins for 24 h, and after incubation, viability was determined employing the MTT assay.

Tepary bean lectin presented a dose-depending cytotoxic effect on the cell clone (evaluated using the MTT test). The cytotoxicities of cell clones were different as shown by the IC_50_ values obtained for each cell clones. For clone T4, we found an IC_50_ of 51.5 µg/mL, for N5, of 72.5 µg/mL, and for J20, of 275 µg/mL. Figure 8 demonstrates that the T4 clone showed significant growth inhibition, while J20 clone showed low inhibition even at a high lectin concentration. Cytotoxicity studies reported for the same lectin [51] have demonstrated that PAA have the ability to inhibit the growth of human cancer cells, either by causing cytotoxic or anti-proliferation effects on SW480 and C33-A cell lines, and, on the other hand, that PAA has the ability to inhibit the colony formation ability in both cell lines. On the other hand, it has also been reported that tepary bean lectin can reduce the viability of small intestine epithelial cells of rats [52], as well as induce severe damage in mice [53].

## 3. Experimental

### 3.1. Materials

White tepary bean (*Phaseolus acutifolius*) seeds were purchased from a local market in Hermosillo (Sonora, Mexico). The beans were ground into a fine powder with a Thomas-Willey mill prior to the lectin isolation procedure. Reagents for protein extraction and purification were purchased from Sigma Chemical Co. (St Louis, MO, USA). Mini-Leak agarose matrix (medium density) was purchased fromKem-En-Tec A/S (Copenaghen, Denmark); Fractogel Azlactone affinity matrix was purchased from Merck (USA). Electrophoresis reagents were acquired from Bio-Rad (Hercules, CA, USA). Erythrocytes of human blood groups A and O were obtained from healthy volunteer donors. The erythrocytes were separated from plasma by centrifugation at 2,000 rpm for 10 min; the cells were then washed twice with two volumes of PBS (10 mM KH_2_ PO_4_/K_2_HPO_4_ 50 mM NaCl, and 15 mM sodium azide, pH 7.2). For agglutination assays, 250 μL of erythrocytes were treated with trypsin (0.6 mg/mL) in 10 mL of PBS for 1 h at 37 ºC and then washed three times with PBS. The erythrocytes were diluted (2%) in PBS before use. Glycoproteins employed in hemagglutination inhibition assays (bovine fetuin, bovine fibrinogen, porcine thyroglobulin, conalbumin, and ovoalbumin) were from Sigma. Recombinant soluble β-1,6-*N*-acetylglucosaminyl transferase V (GnT-V) was purified from overproducer Chinese hamster ovary cells as described [43]. Glycopeptides utilized in hemagglutination and affinity chromatography studies were prepared from the glycoproteins mentioned previously by treating them with trypsin or thermolysin using the procedure reported by Altmann *et al.* [49]. Oligosaccharide mixture from chitin was prepared as described by Merkle and co-workers [54]. Human milk oligosaccharide mixture was a kind gift from David F. Smith (University of Georgia). RPMI 1640 culture media was purchased from Gibco BRL. Mouse 3T3 fibroblast cells clones (T4, N5, and J20) were a gift from Enrique Pérez-Cárdenas, M.Sc. (National Institute of Cancer Research, Mexico). All other reagents were of the highest quality available.

### 3.2. Preparation of Mini-Leak Agarose Resin

Bovine fetuin was immobilized in Mini-Leak agarose by adding 1 g of the the resin (previously washed with de-ionized water) to 50 mg of the protein in 1 mL of 0.1 M NaCl. Polyethyleneglycol was added immediately to a concentration of 20% and the coupling reaction mixture was incubated overnight at room temperature under constant agitation. After incubation, the mixture was settled, the supernatant was decanted, and the resin washed twice with 20 mL of 0.1 M NaCl to remove the uncoupled fetuin. To block the non-reacted active groups, the resin was incubated for 3 h with two volumes of 0.2 M ethanolamine at room temperature. Then the solution was decanted and the resin was packed into an empty chromatography column (1.5 cm × 15 cm) (Bio-Rad) and connected through a flow adapter to an FPLC chromatography system (Amersham Pharmacia Biotech, Uppsala, Sweden). The resin was washed with 20 mL of 0.1 M glycine, pH 2.5, then with 20 mL of 0.1 M of dibasic potassium phosphate, pH 11.0, and finally with 20 mL of 0.1 M NaCl, 15 mM NaN_3_.

### 3.3. Purification of PAA

Purification of PAA was carried out with modification of the method by Mejia *et al.* [37]. Presence of the lectin along the purification procedure was verified by positive hemagglutination in the activity assays described later. Ground tepary beans were extracted overnight with PBS (10 mL/g of bean meal) at 4 ºC in a glass beaker with constant stirring. The mixture was centrifuged at 12,000 g for60 min to yield the crude extract. The crude extract was dialyzed overnight against PBS at 4 ºC and centrifuged again to remove insoluble residues. The extract was then subjected to a “salting out” step with 70% NH_4_SO_4_ (w/v) and centrifuged at 12,000 g for 60 min. The precipitate was resuspended and dialyzed against three changes of PBS. The dialyzed fraction was subjected to affinity chromatography. Prior to sample injection, the affinity column was equilibrated with 20 volumes of PBS. Then the sample was applied and the unbound proteins were washed with 20 volumes of the initial buffer. Fractions that exhibited hemagglutination activity were eluted from the column with 20 volumes of 50 mM glycine-HCl, pH 2.5. The elutes were dialyzed against distilled water with three changes, then lyophilized and stored at −20 °C until further characterization. Protein was determined according to Lowry *et al.* [55] using bovine serum albumin as standard.

### 3.4. Size Exclusion Chromatography

Molecular weight of the purified lectin from tepary beans was estimated by Size exclusion chromatography (SEC) on TSK 3000 SW size exclusion HPLC column (TosoHaas, Japan) that was previously equilibrated in PBS at a flow rate of 0.5 mL/min. An aliquot of the pure lectin (0.1 mg) was dissolved in PBS and injected into the column. The material was eluted from the column and monitored in an online Ultraviolet (UV) detector at wavelength of 280 nm. Molecular weight of the sample was estimated by graphic interpolation on a standard curve (data not shown) obtained by graphing the retention time *vs.* the logarithm of the molecular weight of the following protein standards: thyroglobulin (660 kDa); ferritin (440 kDa); catalase (220 kDa); aldolase (150 kDa); bovine serum albumin (67 kDa), and ovoalbumin (44 kDa). In order to evaluate the role of ionic strength of the mobile phase in the retention time of PAA in SEC, a second system was employed in which this lectin was injected to a Superose 12 column (Amersham Biosciences) under two buffer conditions: the first with 25 mM MES pH 6.0 (low ionic strength); and the second with the same buffer, added together with 1 M NaCl (high ionic strength). Molecular weight in these runs was estimated as described previously.

### 3.5. MALDI-TOF Mass Spectrometry

Molecular weight of the purified tepary bean lectin was also measured by Matrix Assisted Laser Desorption/Ionization-Time of Flight Mass spectrometry (MALDI-TOF MS) (Hewlett Packard-G2025A LD-TOF). The pure lectin was dissolved in 500 μL of water and a 5-μL aliquot of the protein was mixed with 5 μL of sinapinic acid. One to two microliters of sample were then applied to the surface of a gold probe, dried under vacuum, and applied to the spectrometer.

### 3.6. Polyacrylamide Gel Electrophoresis (PAGE)

The purified lectin was subjected to analysis by native PAGE using a Phast System Instrument (Amersham Biosciencies) in 8–25% PhastGel and run as described by the manufacturer (Separation Technique File Number 120). For dissociation of the subunits, SDS-PAGE was carried out according to Laemmli (1970) [56] under reducing conditions in a 12% gel. SDS-PAGE was performed in a vertical miniprotean II electrophoresis system (Bio-Rad). Separated proteins in the gels were visualized by staining with Coomassie blue R-250. Molecular mass standards in native PAGE were from Amersham Biosciences, and in SDS PAGE, were myosin, β-galatosidase, phosphorylase-B, bovine serum albumin, ovalbumin, carbonic anhydrase, and soybean trypsin inhibitor.

For two dimensional-PAGE, Isoelectric focusing (IEF) electrophoresis was performed as the first dimension as described in [57,58]. The line on which the lectin was found was excised from the gel and placed at the top of a 12% SDS-PAGE gel, then sealed with 1% agarose, and then resolved for the second dimension. Proteins separated in the 2D-Gel were detected by silver nitrate staining [59] and scanned in a densitometer (BioRad GS700 Imaging densitometer).

### 3.7. Amino Acid Composition Analysis

Analysis of amino acid composition was performed hydrolyzing the purified lectin and transforming the released amino acids into their fluorescent OPA derivatives, which were then analyzed by reverse phase HPLC according to the method of Vázquez-Ortiz *et al.* [60].

### 3.8. Metal Composition Analysis

The lectin was dissolved with NaCl (0.5%) and subsequently dialyzed against 0.5% NaCl for 12 h, and then against 50 mM EDTA for another 12 h. The dialyzed lectin was digested with nitric acid in a microwave oven (Perkin Elmer) and cations were detected by a plasma spectrometer (plasmaICP-OES, optima 3000XL) [61]. A calibration curve was prepared using standards of each cation (Ca, Cu, Cr, Cd, Fe, Mg, Mn, Zn) (Perkin Elmer) and the concentration of each cation in the lectin sample was estimated by graphic interpolation in ppb.

### 3.9. Carbohydrate Content

Total carbohydrate content of the lectin was estimated by the phenol sulfuric acid method ofDubois [62] utilizing glucose as a standard.

### 3.10. Analysis of Partial Sequences of Peptides Subunits by LC/MS/MS

The subunits of the pure lectin from tepary beans were resolved by 2D-PAGE as described previously and stained with a fluorescent dye (Amersham Biosciences). The 2D gel was placed in a robotized-system Ettan Spot Handling Workstation (Amersham Biosciences) and sections of the gel corresponding to the resolved subunits were excised. These gel sections were digested with trypsin and injected into a microbore C-18 column coupled via a nano-electrospray interface to a quadrupole-Time of Flight mass spectrometer operating in MS/MS mode [63,64]. Mass spectrometry data was analyzed by MASCOT (http://www.matrixscience.com). Deduced amino acid sequences were obtained using the ExPASy Proteomics Server available at (http://www.expasy.gov). Multiple alignments of known protein sequences were produced with the CLUSTAL W program (http://www.ebi.ac.uk/Tools/msa/clustalw2/).

### 3. 11. Hemagglutination Assays

Hemagglutination tests were carried out in microtiter 96-well (U shaped) plates. The lectin was diluted serially (2-fold), adjusting the sample volume in each well to 50 μL with PBS. Diluted samples were each mixed with 50 μL of the 2% suspension of trypsinized human erythrocytes types A and O. The reaction mixture was incubated for 1 h at room temperature and then observed for positive agglutination. The titer was defined as the reciprocal of the highest dilution showing detectable agglutination [65].

For hemagglutination inhibition assays, a PAA solution (50 μL, 0.08 μg/mL) was added to a battery of potential haptens (sugars, oligosaccharides, glycopeptides, and glycoproteins listed in Table 1, 50 μL), each diluted serially (2-fold) with PBS in microtiter plates, incubated for 1 h, and then added with 50 μL of a trypsinized 2% suspension of human erythrocytes. After incubation of the mixture at room temperature for 1 h, the reaction was observed for the end-point of minimum agglutination as described previously. Inhibitory activity was expressed as the lowest concentration of sample solution at which inhibition of hemagglutination was observed.

### 3.12. Preparation of Fractogel-Azlactone-Lectin Matrix

Purified tepary bean lectin was coupled wit Fractogel-Azlactone matrix by adding the resin (290 mg, 1 mL) to the lectin (10 mg) previously suspended in a buffer (2 mL) that contained 0.05 M of KH_2_PO_4_ and 0.6 M sodium citrate, pH 7.5. The reaction mixture was incubated for 4 h at room temperature under constant agitation. The coupling was stopped by adding 500 μL of 3 M ethanolamine for 3 h. Then the solution was decanted and the resin was washed with TBS (20 mL, Tris 50 mM, pH 8.0, NaCl 150 mM, CaCl_2_ 10 mM, and 0.2 mM MgCl_2_). Finally, the matrix was packed in an empty chromatography column (1 × 10 cm) and equilibrated with TBS.

### 3.13. Characterization of Lectin Affinity

PAA immobilized in an azlactone-polyacrylamide affinity resin (2 mL, see previously) was packed into a 1 × 10 cm chromatography column. This column was connected with a flow adaptor to an Akta Purifier 100 chromatography system (Amersham Biosciences) and equilibrated with 10 volumes of TBS. We suspended trypsinized glycopeptides (5.4 mg) from thyroglobulin in the buffer (1.5 mL) and applied this to the affinity column at a flow rate of 1 mL/min. Then the column was washed with 10 volumes of TBS to recover the unbound and retarded material. Then, five volumes of an acidic buffer (50 mM glycine, 150 mM NaCl, pH 2.5) were applied to wash out any bound material from the affinity column. The glycopeptides that were washed and eluted from the affinity column were detected with an online UV detector at 214 nm and collected as 1-mL fractions in glass tubes. Fractions were pooled as unbound, retarded, or bound glycopeptides. These pooled fractions were desalted through a Sephadex G-15 gel filtration column using a volatile buffer (100 mM NH_4_HCO_3_) and dried under reduced pressure. The desalted glycopeptides were then analyzed for sugar composition by TMS derivatization followed by gas chromatography [66].

### 3.14. Mitogenic Activity on Human Lymphocytes

Lymphocytes were obtained from human peripheral blood by centrifugation over a Ficoll gradient (Sigma Chemical Co.). The isolated lymphocytes were diluted 2-fold with PBS, then washed in the same buffer, and finally the cells were suspended in RPMI 1640 culture media containing 10% fetal bovine serum and 1% antibiotic (streptomycin and sodium penicillin G).

The cells were cultured in flat-bottomed microtiter plates at a concentration of 2 × 10^5^ cells/well. Aliquots of pure PAA were added at different concentrations (0, 0.5, 1.0, 5.0, 10.0, 25.0, and 50.0 μg/mL) in medium with a total volume of 200 μL. Incubation was carried out at 37 ºC in an atmosphere of air containing 4% CO_2_ (v/v). After the cells were cultured for 42 h, they were added to 3 μci/mL of [^3^H]thymidine (Amersham Biosciences); then, the culture was incubated for 6 additional hours [32].

After incubation, the cells of each well were poured into a microcentrifuge tube and centrifuged for 10 min at 10,000 rpm to stop uptake of radioactivity, the medium was removed, and the cells were washed twice with PBS; then, 0.1% SDS (500 μL) containing 10 mM EDTA was added. After 20 min at room temperature, the lysate was added to 10% cold trichloroacetic acid (TCA, 500 μL). The precipitate was collected on a nitrocellulose filter (Bio Rad), washed with 3 mL of 5% TCA, and dried, and the filter was dipped into a 4-mL cocktail in a scintillation vial and radioactivity was counted in a liquid scintillation counter (Beckman LS 6500) [67]. All determinations were done in quintuplicate.

### 3.15. Cytotoxicity Activity on Mouse 3T3 Fibroblast Cell Clones

In this assay, we employed three clones of mouse 3T3 fibroblasts previously prepared in the laboratory of Dr Alejandro García-Carrancá; the clones comprised J20, T4, and N5. The cells were cultured in DMEM culture media (Gibco BRL) containing 10% fetal bovine serum and 1% antibiotic (streptomycin and sodic penicillin G) in flat-bottomed microtiter plates at a concentration of1 × 10^4^ cells/well. Aliquots of pure PAA were added at different concentrations (0, 1.0, 10, 100, 200, and 300, μg/mL) in medium with a total volume of 200 μL. Incubation was carried out at 37 ºC in an atmosphere of air containing 4% CO_2_ (v/v) for 24 h. After incubation, the cells were washed and added to culture medium and viability was determined employing the MTT technique [68].

## 4. Conclusions

In this study, it was possible to purify the lectin from *Phaseolus acutifolius* by affinity chromatography using fetuin. However, inhibition tests showed that the lectin can be purified with other glycoproteins, such as thyroglobulin, which shows residues of fucose, mannose, galactose, and *N*-acetyl glucosamine, as analyzed in this study. Thus, PAA could be classified as a complex lectin. PAA is a tetrameric glycoprotein that can behave as a dimer due to the ionic strength of the medium, composed of at least three isoforms, whose subunit molecular weights differed slightly, possibly due to glycosylation differences in their structure. Partial amino acid sequences from PAA had high identity with phytohemagglutinin of *Phaseolus vulgaris* and *Phaseolus coccineus*, showing the need to conserve these molecules. Because the PAA showed toxic effects on human lymphocytes and mouse fibroblasts, the results open the possibility of using this lectin in toxicity studies on normal and transformed cells from human and animal sources. A deeper characterization of isolectins in PAA is required in order to define fine carbohydrate specificities and structural and biochemical differences. Therefore, understanding the structure and function of the lectin would be useful in studies with bacteria, fungi, viruses, and tumor cells, which cause damage to plants and to humans.
